# Evaluation of dietary acidifying and phytogenic additives on performance, carcass characteristics, immune organs, intestinal morphometry, and fecal oocyst shedding in broiler chickens challenged with *Eimeria spp*

**DOI:** 10.1016/j.vas.2026.100933

**Published:** 2026-11-04

**Authors:** Lucas Nunes de Melo, Adiel Vieira de Lima, Apolônio Gomes Ribeiro, Raiane dos Santos Silva, Amanda Itoh de Medeiros, Maria Isabelly Leite Maia, Aline Beatriz Rodrigues, Humberto de Araújo Brito Filho, Edijanio Galdino da Silva, Isabelle Naemi Kaneko, Matheus Ramalho de Lima, Ricardo Romão Guerra, Leonardo Augusto Fonseca Pascoal, Lucas Rannier Ribeiro Antonino Carvalho, Fernando Guilherme Perazzo Costa

**Affiliations:** a Federal University of Paraíba, Department of Animal Science, Areia, Paraíba, Brazil; b Federal University of Paraíba, Department of Veterinary Sciences, Areia, Paraíba, Brazil; c Federal University of Rondônia, Department of Animal Science, Rondônia, Brazil; d Federal Rural University of the Semi-Arid, Department of Animal Science, Mossoró, RN, Brazil; e Federal University of Paraíba, Animal Science Department, Bananerias-PB, Brazil; f Karolinska Institutet, Department of Physiology and Pharmacology, Biomedicum 5B, Solnavägen 9, S-171 77, Stockholm, Sweden

**Keywords:** Short-chain fatty acids, Essential oils, Nutritional strategies, Gut integrity, Coccidiosis

## Abstract

This study evaluated the effects of dietary microencapsulated sodium butyrate (SB) and a phytogenic additive (PA) blend containing oregano, thyme, and cinnamon essential oils on productive performance, intestinal health, and oocyst shedding in broiler chickens challenged with *Eimeria spp*. A trial was conducted using 1280 Cobb 500 chicks from 1 to 40 days of age in a completely randomized design with seven treatments and eight replicates per treatment. Treatments included a negative control (no additives, no challenge) and a 2 × 3 factorial arrangement with or without PA (0.010%) and three SB levels (0.02, 0.03, and 0.05%), with all factorial groups challenged on day 2 with a live attenuated vaccine against *Eimeria spp*. Zootechnical performance and carcass characteristics showed no significant differences among groups. Birds supplemented with 0.03 and 0.05% SB showed reduced relative weight of the bursa of Fabricius (P = 0.009). Duodenal villus height increased in birds receiving PA combined with 0.05% SB (P = 0.0004), while villus-to-crypt ratio remained above 5 in all intestinal segments. On day 10, supplementation with 0.03% SB, alone or combined with PA, significantly reduced oocyst shedding (P = 0.0019). In conclusion, microencapsulated SB, alone or combined with PA, successfully maintained productive performance and carcass traits, while beneficially modulating intestinal morphology and bursa weight in broilers challenged with *Eimeria spp*. Supplementation with 0.03% SB, with or without PA, reduced oocyst shedding.

## Highlights

• Microencapsulated sodium butyrate reduced oocyst shedding at day 10.
• Phytogenic additive enhanced duodenal villus height with butyrate.
• Intestinal morphology preserved under Eimeria challenge (VH >5).
• No effects on growth performance or carcass traits.
• Butyrate supports gut health without impairing broiler performance.

## 1 Introduction

Dietary additives serve a pivotal function in poultry nutrition through the promotion of gastrointestinal wellness and zootechnical efficacy. Within this category, organic acids and phytogenic substances have gathered growing attention as viable substitutes for antimicrobial growth promoters (AGPs) in broiler husbandry.

Historically, antibiotic molecules have been extensively utilized in livestock farming for disease prevention and therapy. Furthermore, subtherapeutic dosages of specific antibiotics were routinely blended into feeds as AGPs to favorably adjust gut microbial ecosystems, enhance feed conversion, and boost overall production parameters (Abd El-Hack et al., 2021; Swelum et al., 2021). Nevertheless, widespread AGP administration has sparked significant public health apprehensions regarding the emergence and propagation of antibiotic-resistant bacteria, potential transmission to human populations, and chemical residue deposition in poultry meat (Callaway et al., 2021). These health concerns prompted regulatory authorities globally to restrict or ban subtherapeutic antibiotic feed additives. In Brazil, the Ministry of Agriculture, Livestock and Supply enforced strict bans on multiple antimicrobial agents previously authorized as growth enhancers.

Nevertheless, withdrawing AGPs from commercial formulations frequently compromises flock growth rates (Puvača et al., 2019a; Reda et al., 2020; Sheiha et al., 2020, 2021a), driving extensive research into alternative dietary options. Despite recent technological progress, studies investigating non-antibiotic feed additives capable of effectively alleviating severe enteric conditions, such as coccidiosis and necrotic enteritis in broilers, remain limited.

Coccidial infections represent a primary trigger for necrotic enteritis onset in broiler flocks (Prescott et al., 2016). Eimeria invasion stimulates excessive mucosal mucus secretion, creating a favorable niche for Clostridium perfringens multiplication—the primary pathogen behind necrotic enteritis, which induces substantial economic damages worldwide. To counter the adverse outcomes associated with antibiotic withdrawal, the poultry sector is actively evaluating functional feed ingredients, including organic acids and essential oils.

Organic acids mediate diverse physiological advantages, encompassing direct bactericidal actions, stress mitigation, villus architecture maturation, immune response tuning, and metabolic fuel provision for enterocytes, ultimately optimizing bird health and performance (Pham et al., 2022). Supplementing poultry feeds with organic acids has been demonstrated to improve nutrient digestibilities, foster beneficial microbiota (Lactobacillus spp.), and constrain pathogenic populations including C. perfringens, *Escherichia coli*, and Salmonella spp. across the enteric tract (Dai et al., 2021). Moreover, their targeted antimicrobial potency against pathogenic challenge agents like *E. coli*, Salmonella spp. (Kazempour & Jahanian, 2017), and C. perfringens (Geier et al., 2010) fortifies immunocompetence and preserves mucosal barrier integrity.

Essential oils represent natural, volatile, aromatic lipophilic extracts obtained from plant structures such as blossoms, buds, seeds, leaves, stems, bark, herbs, roots, or fruits (Brenes & Roura, 2010). They have gained substantial academic interest as feed additives owing to their potent antimicrobial, antiviral, radical-scavenging, and antiparasitic capabilities (Abd El-Hack et al., 2022). Dietary essential oil incorporation can enhance broiler growth metrics by stimulating endogenous digestive enzyme secretion, suppressing pathogenic microflora, lowering unwanted fermentation metabolites, improving nutrient retention, and upregulating antioxidant defenses alongside immune responses (Badr et al., 2026; Fathi et al., 2025a, 2025b, 2025c; Fathi & Zarrinkavyani, 2026; Shazr et al., 2026).

During Eimeria challenge, dietary inclusion of sodium butyrate and phytogenic compounds may support gut health and alleviate coccidiosis-induced damage. Sodium butyrate serves as a vital oxidative substrate for epithelial enterocytes, stimulating cellular multiplication, maintaining structural integrity, and modulating gut microbial composition. In broilers subjected to Eimeria infection, microencapsulated butyric acid administration accelerated mucosal regeneration and structural recovery post-challenge, albeit with modest impacts on live performance (Ventura et al., 2021). Likewise, microencapsulated sodium butyrate application during coccidial challenge influenced intestinal cellular turnover rates and protected mucosal tissue (Lima et al., 2023), while lipid-coated sodium butyrate partially re-established cecal bacterial equilibrium following Eimeria tenella infection (Zhou et al., 2017). Concurrently, phytogenic products formulated with essential oils (notably oregano extracts) promote gastrointestinal health via antimicrobial, antioxidant, and anti-inflammatory pathways. In Eimeria-infected broilers, oregano-derived essential oils decreased oocyst excretion and altered luminal metabolite profiles, whereas combinations of oregano and clove oils partially attenuated challenge-induced gut barrier breakdown (Gordillo Jaramillo et al., 2021; Zhang et al., 2023).

Notwithstanding these findings, comprehensive investigations into the application of microencapsulated sodium butyrate, applied individually or combined with phytogenic additives in *Eimeria* spp. broilers, remain scarce. Specifically, the physiological consequences of combining microencapsulated sodium butyrate with a targeted blend of oregano, thyme, and cinnamon essential oils under coccidial challenge have not been thoroughly elucidated. Additionally, evaluating microencapsulated sodium butyrate in infected birds offers deeper insights into gut morphometry dynamics, live performance outcomes, and oocyst excretion kinetics. Evaluating these additive combinations independently and concurrently will clarify their efficacy as practical nutritional interventions for broilers facing coccidial stress.

Consequently, this study was designed to evaluate the impacts of dietary microencapsulated sodium butyrate supplementation, provided alone or alongside a phytogenic blend of oregano, thyme, and cinnamon essential oils, on gut morphometrics, fecal oocyst shedding, growth performance, carcass yields, and lymphoid organ weights in broilers challenged with *Eimeria spp*. from 1 to 40 days of age.

## 2 Materials and methods

### 2.1 Location and ethics approval

The experimental trial was carried out at the Poultry Sector of the Department of Animal Science, Center for Agricultural Sciences, Federal University of Paraíba (UFPB), Areia, Paraíba, Brazil. All animal handling and experimental protocols received formal approval from the Institutional Animal Ethics Committee (CEUA/UFPB; Approval Protocol No 8214,170,122).

### 2.2 Animals and experimental design

A total of 1280 day-old male Cobb 500 broiler chicks were monitored over a 40-day rearing period. Chicks were randomly distributed into 80 floor pens (20 birds per experimental pen) following a completely randomized arrangement with seven experimental treatments and eight replicates each. Animals were maintained in a open-sided poultry house equipped with reusable floor litter. Throughout the trial, feed and fresh water were offered for ad libitum consumption. The photoperiod program adhered strictly to Cobb 500 Broiler Management guidelines, providing continuous lighting (24 h of combined natural and supplementary light) on day 1, followed by standard management lighting schedules across the remaining rearing period (days 1 to 40).

### 2.3 Diets and experimental treatments

The dietary treatments consisted of a control diet and a 2 × 3 factorial arrangement, with or without supplementation of a phytogenic additive (PA) (Phyto 100®) and three inclusion levels of 30% microencapsulated sodium butyrate (SB) (Table 1).

**Table 1:** Description of the treatments.

Treatments	Supplementation in the experimental diet	Health challenge⁎
NC^1^	Free of performance-enhancing additives	No
0.02% SB	0.00% PA⁎ + 0.02% SB⁎	yes
0.03% SB	0.00% PA + 0.03% SB	yes
0.05% SB	0.00% PA + 0.05% SB	yes
0.010% PA + 0.02% SB	0.010% PA + 0.02% SB	yes
0.010% PA + 0.03% SB	0.010% PA + 0.03% SB	yes
0.010% PA + 0.05% SB	0.010% PA + 0.05% SB	yes

⁎ PA – phytogenic additive, Phyto 100®. ^⁎⁎^SB – microencapsulated sodium butyrate, Sodium Butyrate® Health challenge performed with attenuated vaccine against coccidiosis (Bio-Cocivet). ^1^Negative control.

The experimental diets were formulated by gradually replacing the inert ingredient with the phytogenic additive and sodium butyrate according to the proposed inclusion levels.

The diets were formulated to be isonutritive and isoenergetic, based on corn and soybean meal, according to the nutritional recommendations of Rostagno et al. (2017) for high-performance male broiler chickens. The experimental period was divided into four feeding phases: 1–7, 8–21, 22–35, and 36–40 days of age. The ingredient composition of the experimentais diets for each feeding phase is presented in Table 2. No antibiotics, coccidiostats, or antimicrobial growth promoters were included in the experimental diets. The complete ingredient and nutritional composition of the diets is presented in Table 2.

**Table 2:** Composition of experimental diets for broilers during the experimental period.

Ingredients	Growth Phases			
	1 to 7 days	8 to 21 days	22 to 35 days	36 to 40 days
Corn	527.10	534.90	542.85	638.30
Soybean Meal	395.00	384.25	365.00	291.70
Soybean Oil	30.00	40.50	54.00	35.70
Limestone	10.00	10.50	8.35	7.20
Dicalcium phosphate	20.00	14.70	15.00	10.95
Salt	4.70	3.00	3.60	3.40
L-Lysine HCl	3.50	2.90	2.65	5.48
DL-Methionine	3.90	3.70	3.40	2.70
L-Threonine	1.40	1.30	1.10	0.790
L-Valine	1.10	0.950	0.750	0.480
Choline Chloride	0.700	0.700	0.700	0.700
Premix^1^	1.00	1.00	1.00	1.00
Sodium bicarbonate	1.00	1.00	1.00	1.00
Inert	0.600	0.600	0.600	0.600
Total	1000	1000	1000	1000
Chemical composition				
Metabolizable energy (Mcal/kg)	3031	3,0995	3,1972	3,2005
Crude protein (%)	23.2599	22.7413	21.86	19.5293
Calcium (%)	1.0222	0.9068	0.8257	0.6605
Available phosphorus (%)	0.4873	0.3877	0.3903	0.3091
Sodium (%)	0.2296	0.1885	0.2112	0.2009
Chloride (%)	0.3105	0.2094	0.245	0.2375
Potassium (%)	0.8852	0.8675	0.8345	0.7282
Digestible arginine (%)	1.4343	1.4035	1.3475	1.1493
Digestible isoleucine (%)	0.8957	0.8768	0.8419	0.726
Digestible leucine (%)	1.7792	1.7512	1.6971	1.5542
Digestible lysine (%)	1.3775	1.3052	1.2388	1.2917
Digestible methionine (%)	0.7005	0.6755	0.635	0.5379
Digestible methionine + cystine (%)	0.9962	0.9668	0.9184	0.7988
Digestible phenylalanine (%)	1.0199	0.9999	0.9626	0.3091
Digestible threonine (%)	0.8917	0.8676	0.8215	0.7044
Digestible tryptophan (%)	0.2605	0.2545	0.2436	0.053
Digestible valine (%)	1.0399	1.0074	0.9542	0.822

1 Concentration/kg of product: Vit. A - 15 thousand IU, Vit. D3 - 1500,000 IU. Vit. E-WM 15,000; Vit.B1 - 2.0 g, Vit. B2 - 4.0 g Vit. B6 - 3.0 g, Vit. B12 - 0015 g, nicotinic acid - 25 g, pantothenic acid - 10 g; Vit.K3 - 3.0 g, Folic Acid - 1.0 g. Mn - 60 g, Fe - 80 g, Zn - 50 g, Cu - 10 g, Co - 2 g, I - 1 g and Se - 250 mg.

The phytogenic additive (Phyto 100®) consisted of a blend of oregano (*Origanum spp*.), thyme (*Thymus spp*.), and cinnamon (*Cinnamomum spp*.) essential oils, containing 4.5% carvacrol, 6.75% thymol, and 0.3% cinnamaldehyde as active compounds.

The acidifying additive (Sodium Butyrate®) consisted of 30% microencapsulated sodium butyrate, composed of 70% vegetable oil and 30% sodium butyrate, containing 23.7% butyric acid.

The immunological challenge was performed using the attenuated BIO—COCCIVET vaccine, consisting of a concentrated suspension of attenuated oocysts of *Eimeria acervulina, Eimeria maxima* (strains 52, 47, and 159), *Eimeria praecox, Eimeria tenella*, and *Eimeria mitis*. These isolates were obtained from field outbreaks in Brazil and propagated in specific pathogen-free (SPF) chickens.

The vaccine was administered via the drinking water on the second day of age, following the manufacturer's recommendations (1000 vaccine doses diluted in 10 L of water for every 1000 birds). All birds, except those assigned to the negative control (NC) treatment, were challenged.

### 2.4 Data collection and analyzed variables

#### 2.4.1 Zootechnical performance

Growth performance was evaluated at the end of each experimental phase (1–7, 8–21, 22–35, and 36–40 days of age). The evaluated variables included feed intake (FI), body weight gain (BWG), feed conversion ratio (FCR), and livability. Birds were weighed at the beginning and end of each experimental phase to determine performance.

Mortality was monitored daily throughout the experimental period. No mortality was recorded in any of the experimental treatments, resulting in 100% livability in all groups. Livability was calculated by dividing the final number of birds by the initial number of birds in each experimental unit and multiplying the result by 100. Performance data were analyzed cumulatively for the periods of 1–7, 1–21, 1–35, and 1–40 days of age, since birds remained in the same experimental units throughout the study, allowing for the cumulative effects of the dietary treatments.

At 40 days of age, three birds per experimental unit, with body weights close to the pen average, were selected after an 8-h feed withdrawal. The birds were transported to the Experimental Slaughterhouse, individually weighed, electrically stunned, slaughtered by exsanguination, defeathered, eviscerated, and processed for the evaluation of carcass yield, commercial cuts, and abdominal fat deposition.

Carcass yield (excluding viscera, feet, head, neck, and abdominal fat) was expressed as a percentage of live body weight at slaughter. Breast, thigh, and drumstick yields were calculated relative to carcass weight, whereas abdominal fat was expressed as a percentage of live body weight before slaughter.

For the evaluation of lymphoid organs, two birds per replicate, with body weights close to the average of the experimental unit, were selected at 35 days of age after feed withdrawal and euthanized to determine the relative weights of the spleen, thymus, and bursa of Fabricius, expressed as a percentage of live body weight.

#### 2.4.2 Intestinal morphometry and histological procedures

For intestinal morphometry, one bird from each of four replicates per treatment, with body weight close to the average of the experimental unit, was euthanized by cervical dislocation after feed withdrawal at 35 days of age. Samples from the middle portion of the duodenum, jejunum, and ileum were collected, fixed in 10% buffered formalin, routinely processed for histology, embedded in paraffin, sectioned at 5 μm, and stained using the Periodic Acid–Schiff (PAS) technique.

Digital images were captured using a camera coupled to an Olympus BX-40 light microscope for morphometric evaluation of the intestinal mucosa. Villus height and crypt depth were measured using Motic Image Plus 2.0 software, and the villus height-to-crypt depth ratio (VH:CD) was subsequently calculated. Nine measurements were obtained for each variable per bird.

For fecal oocyst counts, approximately 50 g of excreta were randomly collected from each experimental unit at 10, 20, and 30 days of age. Samples were placed in sealed plastic bags, identified, thoroughly homogenized, and refrigerated until analysis. The homogenized samples were diluted tenfold with water and subsequently diluted (1:10) in saturated sodium chloride solution. Oocyst counts were determined using a McMaster counting chamber and expressed as oocysts per gram (OPG) of excreta, according to Hodgson (1970).

### 2.5 Statistical analysis

Statistical analysis was carried out by ANOVA using the SAS GLM procedure (SAS Institute, 2004). The experiment was conducted in a completely randomized design, consisting of a negative control treatment and six treatments arranged in a 2 × 3 factorial arrangement, with two levels of phytogenic additive supplementation (with or without) and three levels of microencapsulated sodium butyrate supplementation (0.02, 0.03, and 0.05%). The statistical analysis of the factorial treatments considered the main effects of phytogenic additive supplementation (PPA), sodium butyrate supplementation (SB), and their interaction (PPA × SB), according to the following statistical model:

Yijk=μ+PPAj+SBk+(PPA×SB)jk+εijk

where: Yijk = observed value of the dependent variable; μ = overall mean; PPAj = fixed effect of phytogenic additive supplementation (with or without); SBk = fixed effect of sodium butyrate supplementation level (0.02, 0.03, or 0.05%); (PPA × SB)jk = interaction effect between phytogenic additive and sodium butyrate supplementation; εijk = random error associated with the observation, assumed to be normally distributed.

The normality of the residuals was assessed using the Shapiro–Wilk test. The negative control treatment, which was not included in the factorial arrangement, was considered separately for comparisons with the experimental treatments. Upon detecting significant main effects or interactions (P < 0.05), factorial treatment means were evaluated via Tukey’s test, whereas Dunnett’s test was applied to compare the negative control against the factorial groups.

## 3 Results

### 3.1 Performance and carcass characteristics

The performance results are presented in Table 3, Table 4, Table 5, Table 6. No significant differences were observed among dietary treatments for the evaluated performance variables in any of the production phases. Likewise, no significant differences were observed among treatments for carcass characteristics (Table 7).

**Table 3:** Performance of broilers from 1 to 7 days fed supplemented diets with additives acidifiers and phytogenics challenged with *Eimeria sp*.

Treatments	BW, day 1	BW, day 7	BWG	FI	FCR	LIV
	(g)	(g)	(g/bird/day)	(g/bird)	(g/g)	(%)
NC	43.42	145.46	9.90	144.69	0.98	95.00
0.02% SB	43.54	146.19	9.76	145.19	0.99	93.75
0.03% SB	43.46	142.92	9.84	146.80	1.02	95.00
0.05% SB	43.52	143.73	9.70	144.94	1.00	95.55
0.010% PA + 0.02% SB	43.56	146.48	9.93	143.42	0.99	96.25
0.010% PA + 0.03% SB	43.43	145.92	9.87	144.57	0.98	97.50
0.010% PA + 0.05% SB	43.49	145.79	9.83	142.12	0.97	95.62
*P-Value*	0.387	0.967	0.925	0.359	0.538	0.278
SEM	0.054	1.194	0.101	2.142	0.014	1.315

SEM = Standard error of the mean; NC = Negative control; BW = Body weight; BWG = Body weight gain; FI= Feed intake; FCR = feed conversion ratio; LIV = Livability; PA = phytogenic additive (Phyto 100®); SB = microencapsulated sodium butyrate (Sodium Butyrate®).

**Table 4:** Performance of broilers from 1 to 21 days fed supplemented diets with acidifying and phytogenic additives challenged with Eimeria sp.

Treatments	BW, day 21	BWG	ADG	FI	FCR	LIV
	(g)	(g)	(g/bird/day)	(g/bird)	(g/g)	(%)
NC	1149.10	1098.41	73.71	1411.08	1.28	93.12
0.02% SB	1159.09	1098.41	74.37	1430.01	1.29	92.34
0.03% SB	1160.36	1105.81	74.45	1413.92	1.28	94.37
0.05% SB	1151.06	1094.71	73.84	1413.74	1.31	92.50
0.010% PA + 0.02% SB	1157.63	1101.18	74.27	1406.73	1.30	94.37
0.010% PA + 0.03% SB	1141.48	1091.82	73.21	1420.96	1.29	95.00
0.010% PA + 0.05% SB	1148.39	1080.29	73.65	1393.60	1.29	90.62
*P-Value*	0.293	0.085	0.291	0.089	0.639	0.717
SEM	11.172	9.558	0.744	10.705	0.009	1.825

SEM = Standard error of the mean; NC = Negative control; BW = Body weight; BWG = Body weight gain; ADG = Average daily gain; FI= Feed intake; FCR = feed conversion ratio; LIV = Livability; PA = phytogenic additive (Phyto 100®); SB = microencapsulated sodium butyrate (Sodium Butyrate®).

**Table 5:** Performance of broilers from 1 to 35 days fed diets supplemented with challenged acidifying and phytogenic additives with Eimeria sp.

Treatments	BW, day 35	BWG	ADG	FI	FCR	LIV
	(g)	(g)	(g/bird/day)	(g)	(g/g)	(%)
CN	2711.18	2588.53	177.84	3766.41	1.45	87.34
0.02% SB	2724.59	2608.76	178.73	3218.40	1.52	76.87
0.03% SB	2695.76	2612.82	176.81	3850.71	1.46	93.12
0.05% SB	2706.85	2597.27	177.55	3849.09	1.47	89.61
0.010% PA + 0.02% SB	2721.03	2613.90	178.51	3823.54	1.48	91.25
0.010% PA + 0.03% SB	2703.17	2589.61	177.32	3814.47	1.47	93.28
0.010% PA + 0.05% SB	2725.75	2589.18	178.81	3788.80	1.45	88.75
*P-Value*	0.974	0.643	0.975	0.743	0.770	0.526
SEM	21.195	28.386	1.555	22.789	0.009	1.940

SEM = Standard error of the mean; NC = Negative control; BW = Body weight; BWG = Body weight gain; ADG = Average daily gain; FI= Feed intake; FCR = feed conversion ratio; LIV = Livability; PA = phytogenic additive (Phyto 100®); SB = microencapsulated sodium butyrate (Sodium Butyrate®).

**Table 6:** Performance of broilers from 1 to 40 days fed diets supplemented with acidifying and phytogenic additives challenged with *Eimeria* sp.

Treatments	BW, day 40	BWG	ADG	FI	FCR	LIV
	(g)	(g)	(g/bird/day)	(g)	(g/g)	(%)
NC	3235.54	3170.28	214.06	4878.49	1.53	76.64
0.02% SB	3319.43	3218.40	219.14	4914.28	1.52	76.87
0.03% SB	3211.98	3151.59	211.23	4865.29	1.54	80.62
0.05% SB	3226.74	3203.46	213.85	4846.93	1.52	78.90
0.010% PA + 0.02% SB	3280.41	3210.58	214.45	4853.05	1.52	79.77
0.010% PA + 0.03% SB	3270.44	3192.81	213.61	4832.77	1.54	81.17
0.010% PA + 0.05% SB	3253.17	3202.57	215.77	4846.45	1.50	76.25
*P-Value*	0.675	0.461	0.311	0.435	0.380	0.515
SEM	40.865	34.283	1.502	44.577	0.032	0.667

SEM = Standard error of the mean; NC = Negative control; BW = Body weight; BWG = Body weight gain; ADG = Average daily gain; FI= Feed intake; FCR = feed conversion ratio; LIV = Livability; PA = phytogenic additive (Phyto 100®); SB = microencapsulated sodium butyrate (Sodium Butyrate®).

**Table 7:** Carcass yield of broilers at 40 days of age, fed diets supplemented with acidifying and phytogenic additives challenged with *Eimeria* sp.

Treatments	Body weight	Carcass yield	Breast yield	Thigh and drumstick yield	Abdominal Fat
	(g)	(%)	(%)	(%)	(%)
NC	3177.16	74.65	41.03	29.18	0.84
0.02% SB	3209.50	74.88	41.38	28.92	0.82
0.03% SB	3142.13	74.88	41.38	29.01	0.74
0.05% SB	3137.99	74.14	41.19	29.01	0.74
0.010% PA + 0.02% SB	3154.26	74.63	41.04	29.24	0.87
0.010% PA + 0.03% SB	3183.17	74.33	40.81	29.14	0.88
0.010% PA + 0.05% SB	3195.24	74.76	41.20	29.51	0.83
SEM	29.455	0.348	0.374	0.322	0.046
*P-Value*	0.51	0.736	0.812	0.453	0.971

SEM = Standard error of the mean; NC = Negative control; PA = phytogenic additive (Phyto 100®); SB = microencapsulated sodium butyrate (Sodium Butyrate®).

### 3.2 Organs immune system

The results for the relative weights of immune system organs are presented in Table 8. No significant effects of dietary treatments were observed on spleen and thymus relative weights. However, the relative weight of the bursa of Fabricius was lower in birds fed diets containing 0.03% SB and 0.05% SB compared with those receiving the NC treatment (P = 0.009).

**Table 8:** Relative weight of lymphoid organs (Spleen, Thymus and Bursa of Fabricius) of broilers at 35 days of age, fed diets supplemented with acidifying and phytogenic additives challenged with Eimeria sp.

Treatments	Body weight	Spleen	Timo	Bursa of Fabricius
	(g)	(%)	(%)	(%)
NC	2861.94^ab^	0.107	0.324	0.086^a^
0.02% SB	2849.91^ab^	0.102	0.330	0.068^ab^
0.03% SB	2807.00^ab^^c^	0.098	0.396	0.062^b^
0.05% SB	2798.30^ab^^c^	0.105	0.327	0.063^b^
0.010% PA + 0.02% SB	2768.36^bc^	0.100	0.346	0.069^ab^
0.010% PA + 0.03% SB	2717.50^c^	0.107	0.347	0.071^ab^
0.010% PA + 0.05% SB	2762.25^bc^	0.121	0.338	0.080^ab^
SEM	25.594	0.006	0.018	0.004
*P-Value*	<0.001	0.126	0.016	0.009

ab Means with different letters in the column differed statistically by Tukey's test at 5% probability. SEM = Standard error of the mean; NC = Negative control; PA = phytogenic additive (Phyto 100®); SB = microencapsulated sodium butyrate (Sodium Butyrate®).

### 3.3 Intestinal morphometry

The results of intestinal morphometry are presented in Table 9. In the duodenum, higher villus heights were observed in birds from the 0.010% PA + 0.05% SB treatment (P = 0.004), lower crypt depths were observed in birds from the NC treatment (P = 0.004), and higher V:C ratios were observed in birds from the CN treatment (P = 0.030). In the jejunum and ileum, no significant differences were observed among dietary treatments for the variables analysed.

**Table 9:** Intestinal morphometry of broilers at 35 days of age, fed diets supplemented with challenged acidifying and phytogenic additives with Eimeria sp.

Treatments	Villus height	Crypt depth	Villus:crypt ratio
	µm	µm	µm/µm
	Duodenum		
NC	1450.491 ^c^	167.428 ^b^	8.75 ^a^
0.02% SB	1662.401 ^bc^	292.250 ^a^	5.92 ^b^
0.03% SB	1773.732 abc	217.201 ^ab^	8.15 ^a^
0.05% SB	1849.079 abc	265.689 ^ab^	7.04 ^a^
0.010% PA + 0.02% SB	2106.864 ^ab^	307.937 ^a^	6.84 ^ab^
0.010% PA + 0.03% SB	1921.806 abc	295.846 ^a^	6.79 ^ab^
0.010% PA + 0.05% SB	2252.486 ^a^	313.939 ^a^	7.21 ^a^
*P-Value*	0.004	0.004	0.030
SEM	47.178	17.880	0.388
	Jejunum		
NC	1627.313	205.601	7.94
0.02% SB	1513.004	208.051	7.26
0.03% SB	1349.290	182.397	7.39
0.05% SB	1406.233	161.684	8.78
0.010% PA + 0.02% SB	1327.170	176.889	7.65
0.010% PA + 0.03% SB	1353.097	178.760	7.85
0.010% PA + 0.05% SB	1113.397	158.143	7.50
*P-Value*	0.188	0.484	0.925
SEM	44.616	13.275	0.567
	Ileum		
NC	1396.453	152.029	9.22
0.02% SB	1150.981	149.665	7.67
0.03% SB	1318.502	143.918	9.22
0.05% SB	1348.082	162.334	8.31
0.010% PA + 0.02% SB	1292.796	148.478	8.80
0.010% PA + 0.03% SB	1270.055	148.093	8.59
0.010% PA + 0.05% SB	1295.207	149.849	8.86
*P-Value*	0.850	0.965	0.691
SEM	48.583	19.29	0.445

abc Means with different letters in the column differed statistically by Tukey's test at 5% probability. SEM = Standard error of the mean; NC = Negative control; PA = phytogenic additive (Phyto 100®); SB = microencapsulated sodium butyrate (Sodium Butyrate®).

### 3.4 Oocyst count in excreta

The results of oocyst counts in excreta are presented in Table 10. On day 10, lower oocyst counts were observed in birds from the 0.03% SB and 0.010% PA + 0.03% BS treatments compared with the NC treatment (P = 0.019). On day 20, higher oocyst counts were observed in birds from the 0.010% PA + 0.02% BS treatment compared with the NC treatment (P = 0.007). No significant differences were observed among the treatments evaluated on day 30.

**Table 10:** Oocyst count in the excreta of broilers at 10, 20 and 30 days of age, fed diets supplemented with acidifying and phytogenic additives challenged with Eimeria sp.

Treatments	Day 10 (oocysts/g)	Day 20 (oocysts/g)	Day 30 (oocysts/g)
NC	12,562.50^a^	2687.50^ab^	5362.50
0.02% SB	5727.50^b^	3785.94^ab^	5150.00
0.03% SB	3200.00^b^	2912.50^ab^	1884.00
0.05% SB	6885.71^b^	6092.19^ab^	5462.50
0.010% PA + 0.02% SB	4028.57^b^	20,881.63^a^	5796.88
0.010% PA + 0.03% SB	3237.50^b^	312.50^b^	3450.00
0.010% PA + 0.05% SB	4214.29^b^	250.00^b^	2050.00
SEM	20.60	52.95	24.52
*P-Value*	0.019	0.007	0.122

ab Means with different letters in the column differed statistically by Tukey's test at 5% probability. SEM = Standard error of the mean; NC = Negative control; PA = phytogenic additive (Phyto 100®); SB = microencapsulated sodium butyrate (Sodium Butyrate®).

## 4 Discussion

At present, identifying functional substitutes for synthetic growth promoters represents a primary focus in poultry research (Alhotan & Abudabos, 2019; Al-Quraishy et al., 2020; Fathi et al., 2025a,b). Beyond demonstrating antimicrobial potency (Du et al., 2016), essential oil administration can favorably influence broiler performance (Namdeo et al., 2020; Fathi et al., 2025a), a trait similarly observed with organic acid supplementation (Polycarpo et al., 2017). Consequently, combining organic acids with essential oils to optimize overall zootechnical indices has gained notable traction within commercial poultry production.

In this trial, growth performance parameters were not significantly enhanced by microencapsulated sodium butyrate inclusion, whether supplied independently or in tandem with essential oils. Comparable lack of performance response in broilers has been documented following dietary inclusion of essential oils (Galli et al., 2021; Jang et al., 2007) as well as organic acids (Cerisuelo et al., 2014). Conversely, several authors have described positive growth benefits when incorporating such feed additives (Du et al., 2016; Eerden et al., 2022; Kumar et al., 2021). Such conflicting results across the literature likely stem from variations in additive formulations, inclusion rates, rearing environment, sanitary status, and challenge severity. In our experimental design, the phytogenic product was tested at a single dose (0.010%), whereas sodium butyrate was evaluated across three concentrations (0.02, 0.03, and 0.05%). Thus, the lack of significant growth response cannot rule out whether alternative inclusion levels or ratio combinations of the phytogenic product might yield distinct physiological responses.

Furthermore, the microencapsulated sodium butyrate product used in the present study contained 30% sodium butyrate, with the remaining 70% consisting of vegetable oil. Microencapsulation may influence the stability, release characteristics, and delivery of the active compound along the gastrointestinal tract, potentially affecting its biological efficacy. However, because the present study did not compare different encapsulation technologies or microencapsulated and non-encapsulated sodium butyrate, the specific contribution of microencapsulation to the observed responses cannot be determined. These considerations highlight the need for further studies evaluating a broader range of phytogenic additive inclusion levels, dose-response relationships, and different butyrate formulations under coccidial challenge conditions.

Apart from modulating gastrointestinal pH, organic acid preparations like butyrate mediate multiple physiological functions in birds. Butyrate acts as a direct metabolic substrate for enterocytes and participates in preserving mucosal barrier tightness, regulating inflammatory pathways, and structuring gut microbial ecology (Ahsan et al., 2016; Melaku et al., 2024). Furthermore, its intrinsic antibacterial capability aids in suppressing enteric pathogens, whereas enhanced epithelial health facilitates enzymatic digestion and nutrient absorption (Ahsan et al., 2016; Naghizadeh et al., 2022). Phytogenic essential oils, on the other hand, carry active phenolic and terpenoid constituents that disrupt microbial cell membranes to exert antibacterial effects, while simultaneously modulating digestive enzyme production and systemic host responses (Fathi et al., 2023, 2025a; Zhai et al., 2018). Combining both additives may therefore trigger additive or synergistic modes of action across distinct biological pathways. However, the extent to which these mechanisms were elicited under the conditions of the present study cannot be established, since gastrointestinal pH, microbial populations, nutrient digestibility, and intestinal barrier function were not directly evaluated. Furthermore, although sodium butyrate was tested at three inclusion levels, the absence of a consistent performance response does not establish whether these levels were sufficient to induce the proposed biological effects. Further dose-response studies incorporating direct measurements of these mechanisms are therefore warranted.

Even though growth traits did not differ statistically, combining organic acids with essential oils could influence flock responses via distinct modes of action and potential complementary pathways. Chapman (2014) pointed out that Eimeria-infected birds typically experience marked weight loss and depressed feed intake, driven by malabsorption and the metabolic burden of mounting an immune defense. In our study, challenged groups exhibited zootechnical performance comparable to the non-challenged control (NC). Nevertheless, because a challenged, unsupplemented control group was not included, it is not possible to determine whether the dietary additives mitigated the potential effects of the Eimeria challenge on productive performance. Therefore, the similarity in performance between the challenged treatments and the NC should not be interpreted as evidence of a beneficial effect of the additives on growth performance or as proof that the effects of the challenge were mitigated.

Dietary treatments did not alter broiler carcass traits. The absence of significant differences in dressing percentage or commercial cut yields aligns with previous studies indicating negligible impacts of dietary organic acids and phytogenics on carcass parameters in broilers (El-Ashram & Abdelhafez, 2020; El-Kasrawy et al., 2023). These findings suggest that, under the experimental conditions of the present study, supplementation with microencapsulated sodium butyrate, alone or combined with essential oils, did not result in measurable changes in carcass traits.

Concerning lymphoid organs, treatment effects were limited to the relative mass of the bursa of Fabricius. Lower relative weights were observed in birds supplemented with 0.03% and 0.05% sodium butyrate compared with the NC group, whereas the other treatments showed similar values. The relative weights of the other immune organs evaluated were not affected by the dietary treatments. Although the bursa of Fabricius is an important organ involved in B-cell development, changes in its relative weight alone are insufficient to establish whether immune function was stimulated, suppressed, or otherwise modulated. Therefore, the lower bursa weight observed in some sodium butyrate-supplemented groups should be interpreted cautiously, as its biological significance cannot be determined from the present results. Further studies involving specific immunological parameters are needed to clarify the effects of sodium butyrate and phytogenic additives on immune function in broilers. Earlier reports have documented that organic acid and phytogenic supplementation can modulate systemic and mucosal immune responses in poultry (Kazempour & Jahanian, 2017; Kumar et al., 2021; Pham et al., 2022).

On the other hand, supplementation with sodium butyrate and phytogenic additives altered intestinal morphology. The small intestine is the segment of the digestive system in birds where the greatest nutrient absorption occurs. The intestinal mucosa has a high absorptive capacity, and it is believed that greater villus development increases the surface area available for nutrient absorption. However, it has also been suggested that longer villi may represent a compensatory mechanism that occurs during challenging periods, when birds require greater nutrient absorption (Galli et al., 2021). Intestinal crypts produce enterocytes that compose the villi, and their depth is related to the synthesis of these cells. Therefore, shallow crypt depth suggests decreased cellular turnover demands, allowing a higher fraction of dietary nutrients and energy to be partitioned into body growth (Lemos et al., 2013).

However, the greater the exposure of villi to lesions caused by pathogens, toxicity, or antinutritional factors, the greater the synthesis of enterocytes by the crypts to promote cell renewal. Consequently, increased crypt depth serves as a marker for accelerated enterocyte proliferation (Sabino et al., 2018). This explains the greater crypt depths observed in the dietary treatments challenged with *Eimeria spp*. compared with the NC treatment, which did not receive the challenge. The villi were likely exposed to lesions caused by the challenge, stimulating enterocyte synthesis by the crypts and increasing cell renewal. In addition, greater crypt depth and reduced villus height are associated with a lower number of absorptive cells and a greater number of secretory cells. Overproduction of mucin by goblet cells coats the mucosal layer and, in excess, can interfere with nutrient transport (Sabino et al., 2018).

Assessing the villus-to-crypt ratio provides an indicator of intestinal functionality, where values above 5 typically reflect a healthy and functional intestinal mucosa (Petrolli et al., 2012). Our results demonstrate that the addition of sodium butyrate alone or in combination with a phytogenic additive was effective because, even though birds were challenged with *Eimeria sp*., intestinal development was not compromised, considering that the V:C ratio was greater than 5 in all segments of the small intestine.

*Eimeria* challenge impairs local immunity and stimulates excessive mucus synthesis, creating ideal conditions for C. perfringens proliferation (Forder et al., 2012), the causative agent of necrotic enteritis, a disease responsible for significant losses in broiler production. In the present study, sodium butyrate supplementation alone and in combination with essential oils reduced oocyst production on day 10 compared with the NC group. Although the negative control (NC) group was not intentionally challenged with the coccidiosis vaccine, oocysts were detected in fecal samples collected at 10 days of age. This finding may be associated with incidental environmental exposure or potential cross-contamination, as all experimental groups were housed in the same poultry house, with pens separated only by wire mesh, which prevented complete physical isolation of the unchallenged birds from the challenged groups. Similarly, the higher oocyst count observed at 20 days of age in birds receiving 0.010% phytogenic additive (PA) combined with 0.02% sodium butyrate (SB) may be related to differences in environmental exposure, the heterogeneous distribution of oocysts within the poultry house, and the inherent variability associated with fecal sampling and oocyst enumeration. However, the specific causes of these findings could not be established. Therefore, these results should be interpreted with caution, as they do not necessarily indicate a specific effect of the dietary treatments on oocyst shedding.

High fecal oocyst shedding serves as a primary risk factor driving coccidiosis outbreaks in commercial poultry units (Wondimu et al., 2019). Therefore, these results indicate that the additives evaluated in the present study may be effective in preventing diseases such as necrotic enteritis and coccidiosis, which are among the main causes of economic losses in broiler production.

## 5 Conclusions

The addition of microencapsulated sodium butyrate, alone or in combination with a blend of oregano, thyme, and cinnamon essential oils, did not compromise productive performance or carcass characteristics and was associated with changes in intestinal morphology in broilers challenged with *Eimeria spp*. The dietary treatments also influenced the relative weight of the bursa of Fabricius; however, the biological significance of this finding remains unclear in the absence of specific immunological assessments. Overall, microencapsulated sodium butyrate, alone or combined with phytogenic additives, may represent a potential nutritional strategy for broilers, although further studies are needed to confirm its effects on intestinal health and immune function under coccidial challenge conditions.

## Ethics statement

The experiment was conducted at the Poultry Module of the Department of Animal Science, Center for Agrarian Sciences, Federal University of Paraíba (UFPB), Areia, Paraíba, Brazil, and all procedures were approved by the Institutional Animal Ethics Committee (CEUA/UFPB; protocol No 8214,170,122).

## Funding

This work was supported by Karolinska Institutet through an institutional publication agreement, which covered the open access publication fees.

## Declaration of generative AI and AI-assisted technologies in the manuscript preparation process

The author(s) declared that generative AI was not used in the creation of this manuscript.

## Data availability

The data are available from the corresponding author upon reasonable request.

## CRediT authorship contribution statement

**Lucas Nunes de Melo:** Writing – review & editing, Writing – original draft, Supervision, Methodology, Investigation, Formal analysis, Conceptualization. **Adiel Vieira de Lima:** Writing – review & editing, Investigation, Formal analysis, Conceptualization. **Apolônio Gomes Ribeiro:** Writing – review & editing, Investigation, Formal analysis, Conceptualization. **Raiane dos Santos Silva:** Writing – review & editing, Investigation, Formal analysis. **Amanda Itoh de Medeiros:** Writing – review & editing, Investigation, Formal analysis. **Maria Isabelly Leite Maia:** Writing – review & editing, Investigation, Formal analysis. **Aline Beatriz Rodrigues:** Writing – review & editing, Investigation, Conceptualization. **Humberto de Araújo Brito Filho:** Writing – review & editing, Investigation, Conceptualization. **Edijanio Galdino da Silva:** Writing – review & editing, Investigation, Formal analysis. **Isabelle Naemi Kaneko:** Writing – review & editing, Investigation, Formal analysis. **Matheus Ramalho de Lima:** Writing – review & editing, Investigation, Formal analysis. **Ricardo Romão Guerra:** Writing – review & editing, Investigation, Formal analysis, Conceptualization. **Leonardo Augusto Fonseca Pascoal:** Writing – review & editing, Investigation, Conceptualization. **Lucas Rannier Ribeiro Antonino Carvalho:** Writing – review & editing, Investigation, Formal analysis, Conceptualization. **Fernando Guilherme Perazzo Costa:** Writing – review & editing, Writing – original draft, Validation, Project administration, Methodology, Conceptualization.

## Declaration of competing interest

The authors declare that they have no other conflicts of interest.
